# Overexpression of thymidylate synthase (TYMS) is associated with aggressive tumor features and early PSA recurrence in prostate cancer

**DOI:** 10.18632/oncotarget.3107

**Published:** 2015-02-25

**Authors:** Christoph Burdelski, Christian Strauss, Maria Christina Tsourlakis, Martina Kluth, Claudia Hube-Magg, Nathaniel Melling, Patrick Lebok, Sarah Minner, Christina Koop, Markus Graefen, Hans Heinzer, Corinna Wittmer, Till Krech, Guido Sauter, Waldemar Wilczak, Ronald Simon, Thorsten Schlomm, Stefan Steurer

**Affiliations:** ^1^ General, Visceral and Thoracic Surgery Department and Clinic, University Medical Center Hamburg-Eppendorf, Germany; ^2^ Institute of Pathology, University Medical Center Hamburg-Eppendorf, Germany; ^3^ Martini-Clinic, Prostate Cancer Center, University Medical Center Hamburg-Eppendorf, Germany; ^4^ Department of Urology, Section for translational Prostate Cancer Research, University Medical Center Hamburg-Eppendorf, Germany

**Keywords:** TYMS, prostate cancer, TMPRSS2-ERG fusion, tissue microarray, prognosis

## Abstract

Thymidylate synthase (TYMS) plays a role in DNA synthesis and is a target for 5-fluorouracil. In this study TYMS was analyzed by immunohistochemistry on a tissue microarray containing 11,152 prostate cancers. TYMS expression was higher in neoplastic than in normal prostate epithelium and was detectable in 72.9% of 10,223 interpretable cancers. It was considered strong in 21.9%, moderate in 33.4% and weak in 17.6% of tumors. TYMS overexpression was associated with deletions at 5q21 (*p* < 0.0001), 6q15 (*p* < 0.0001) and 3p13 (*p* = 0.0083) and gradually increased with the total number of these deletions present in the respective cancer sample (*p* < 0.0001). TYMS expression was unrelated to PTEN deletions (*p* = 0.9535) but tightly linked to high Gleason grade, advanced pathological tumor stage and early PSA recurrence (*p* < 0.0001). The prognostic value of TYMS was independent from the ERG status and deletions at 3p13, 5q21, and 6q15. In multivariate analyses the prognostic role of TYMS expression was independent of Gleason grade, pT stage, preoperative PSA, pN stage, or resection margins. TYMS expression analysis might result in clinically useful information in prostate cancer. The striking link to some but not all chromosomal aberrations might suggest a mechanistical link with specific types of DNA damage.

## INTRODUCTION

Prostate cancer is the most prevalent cancer in men in Western societies [1]. Although the majority of these cancers represent low-malignant tumors with an indolent clinical behavior, some do progress to aggressive and potentially life-threatening disease. Distinguishing between these two appearances of prostate cancer remains difficult [2]. At present, the only established prognostic parameters that are available prior to treatment decisions include Gleason grade and tumor extent on biopsies, prostate-specific antigen (PSA) levels, and clinical stage. Although statistically powerful, these parameters are not sufficient for individual treatment decisions, and overtreatment is a frequent issue for prostate cancer patients. It can thus be hoped that a better understanding of disease biology will eventually lead to the identification of clinically applicable molecular markers that enable a more reliable prediction of prostate cancer aggressiveness.

Thymidylate synthase (TYMS) plays an essential role in the biosynthesis of the DNA-component thymidylate (dTTP) and is required for DNA replication and repair [3]. Accordingly, TYMS expression is a rate-limiting feature for cell proliferation and also for cancer growth [4, 5]. In-vitro studies have shown that overexpression of TYMS is sufficient to transform immortalized mammalian cells to a malignant phenotype [6]. Studies analyzing TYMS expression in cohorts of clinical samples reported a link between TYMS up-regulation and adverse clinical behavior in many solid tumor types including lung [7], breast [8], gastric [9], colorectal [10], and renal cell cancers [11]. Also for prostate cancer, some studies involving 52–172 patients have suggested that TYMS expression levels may be linked to unfavorable tumor phenotype [12–15].

To further evaluate the relevance of TYMS expression in prostate cancer, we took advantage of our preexisting tissue microarray containing >11,000 prostate cancer specimens with clinical follow up and an attached molecular database, and performed an immunohistochemistry analysis of TYMS expression. The results of our study demonstrate that TYMS overexpression is strongly linked to the subset of aggressive prostate cancers characterized by early PSA recurrence and molecular features of chromosomal instability.

## MATERIALS AND METHODS

### Patients

Radical prostatectomy specimens were available from 11,152 patients undergoing surgery between 1992 and 2011 at the Department of Urology and the Martini Clinics at the University Medical Center Hamburg-Eppendorf. Follow-up data were available from a total of 9,695 patients, with a median follow-up of 31.2 months (range: 0.3 to 228 months; Supplementary Table 1). Prostate specific antigen (PSA) values were measured following surgery and PSA recurrence was defined as the time point when postoperative PSA was at least 0.2 ng/ml and increasing at subsequent measurements. All prostatectomy specimens were analyzed according to a standard procedure, including complete embedding of the entire prostate for histological analysis [16]. The TMA manufacturing process was described earlier in detail [17]. In short, one 0.6mm core was taken from a representative tissue block from each patient. The tissues were distributed among 24 TMA blocks, each containing 144 to 522 tumor samples. Each TMA block also contained various control tissues, including normal prostate tissue. The molecular database attached to this TMA comprised results on ERG expression in 9,619 [18], *ERG* break apart FISH analysis in 6,106 (expanded from [19]) and deletion status of 5q21 (*CHD1*) in 7,222 (expanded from [20]), 6q15 (*MAP3K7*) in 3,523 (expanded from [21]), *PTEN* (10q23) in 6,109 (expanded from [22]) and 3p13 (*FOXP1*) in 6,410 (expanded from [21]) cancers. The usage of archived diagnostic left-over tissues for manufacturing of tissue microarrays and their analysis for research purposes as well as patient data analysis has been approved by local laws (HmbKHG, §12,1) and by the local ethics committee (Ethics commission Hamburg, WF-049/09 and PV3652). All work has been carried out in compliance with the Helsinki Declaration.

### Immunohistochemistry

Freshly cut TMA sections were immunostained on one day and in one experiment. Slides were deparaffinized and exposed to heat-induced antigen retrieval for 5 minutes in an autoclave at 121°C in pH 9 Dako Target Retrieval Solution. Primary antibody specific for TYMS (mouse monoclonal antibody (clone 3A1), Abnova, Taipei, Taiwan; cat#H00007298-M01; dilution 1:450) was applied at 37°C for 60 minutes. Bound antibody was then visualized using the EnVision Kit (Dako, Glostrup, Denmark) according to the manufacturer´s directions. The staining intensity (0, 1+, 2+, 3+) and the fraction of positive tumor cells were recorded for each tissue spot. A final score was built from these two parameters according to the following criteria [19]: Tumors with complete absence of staining were scored as “negative”. Tumors scored “weak” had a staining intensity of 1+ in ≤70% of tumor cells or 2+ in ≤30% of tumor cells. A “moderate” score was given to cancers with a staining intensity of 1+ in >70% of tumor cells, or 2+ in >30% and ≤ 70% of tumor cells, or 3+ in ≤30% of tumor cells. The score was “strong” if staining intensity was 2+ in >70% of tumor cells or 3+ in >30% of tumor cells.

### Statistics

Statistical calculations were performed with JPM 9 software (SAS Institute Inc., NC, USA). Contingency table analysis and chi^2^-tests were performed to search for associations between molecular parameters and tumor phenotype. Survival curves were calculated according to Kaplan-Meier. The Log-Rank test was applied to detect significant differences between groups. Cox proportional hazards regression analysis was performed to assess the statistical independence and significance between pathological, molecular and clinical variables. Separate analyses were performed using different sets of parameters available either before or after prostatectomy.

## RESULTS

### Technical aspects

A total of 929 of 11,152 (8.3%) analyzed tissue samples were non-informative due to the complete lack of tissue or absence of unequivocal cancer cells.

### Immunohistochemistry of TYMS in prostate cancer

**Figure 1:**
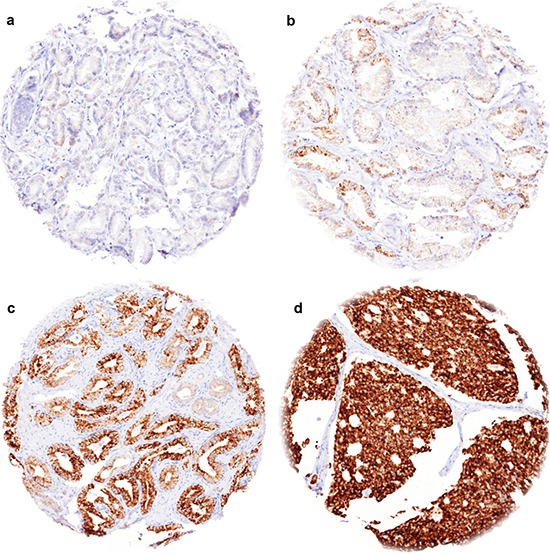
Representative images of TYMS immunostainings in prostate cancer **(a)** negative staining, **(b)** weak staining, **(c)** moderate staining, **(d)** strong staining.

TYMS immunostaining was typically negative and sometimes weak in luminal cells of benign prostate glands, while basal cells were consistently negative. In prostate cancers, cytoplasmic TYMS staining was observed in 72.9% of 10,223 interpretable cancer cases. Detectable TYMS staining was considered weak in 17.6%, moderate in 33.4% and strong in 21.9% of cases. Representative images are given in Figure 1a–1d.

### Association with ERG status

**Figure 2:**
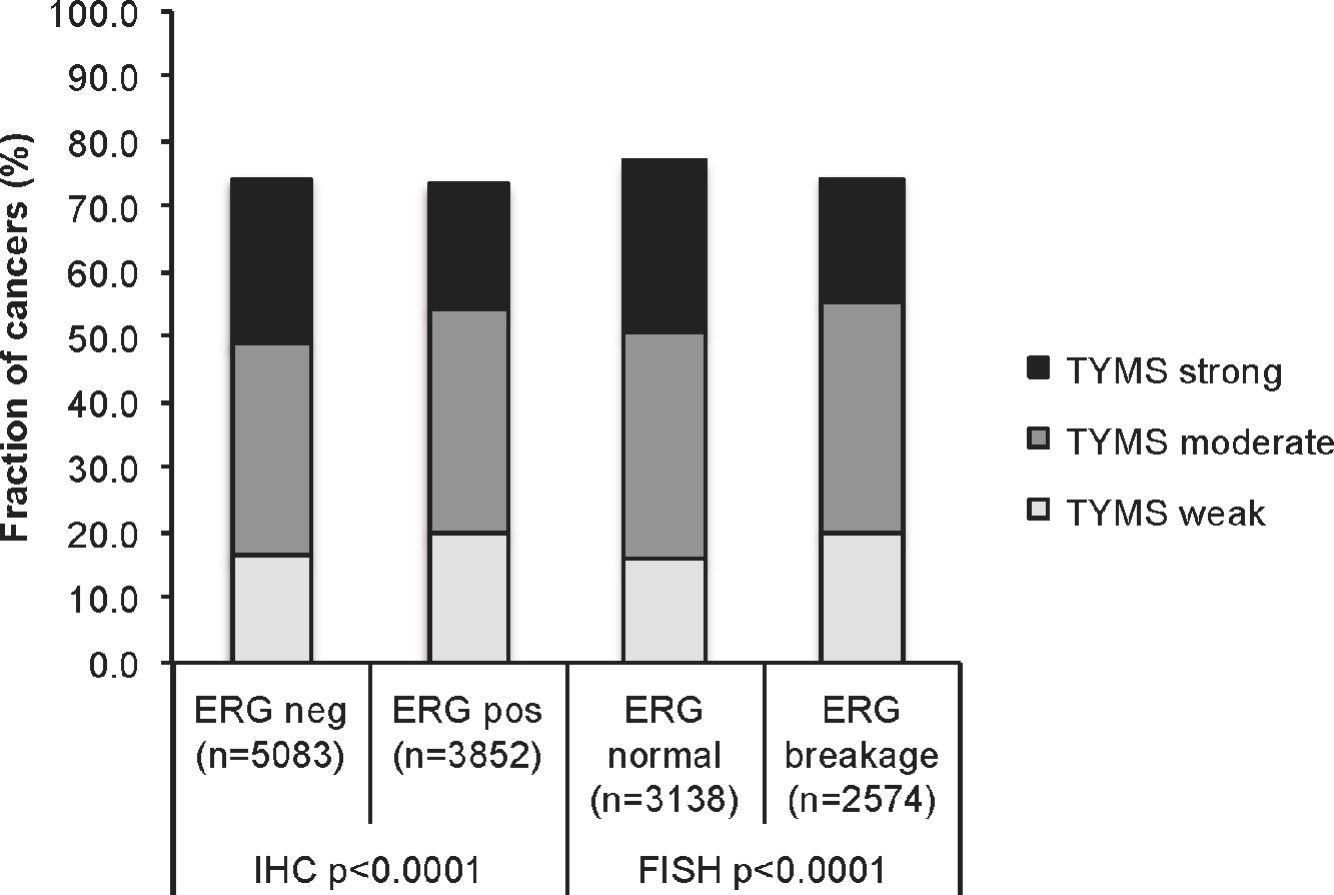
Association between TYMS expression levels and ERG-fusion state Comparison of TYMS expression levels in ERG-positive and ERG-negative prostate cancers. ERG-fusion state was determined either by immunohistochemistry, or by FISH for ERG gene breakage.

Data on both TYMS staining and ERG status obtained by ERG-FISH were available in 5,712 patients and by ERG-IHC from 8,935 tumors. Strong TYMS staining was slightly more frequent in ERG-negative than in ERG-positive tumors both by FISH and by IHC. As the tumor sets were very large, statistical differences between the ERG positive and negative groups were still highly significant (*p* < 0.0001; Figure 2).

### Association with recurrent deletions

**Figure 3:**
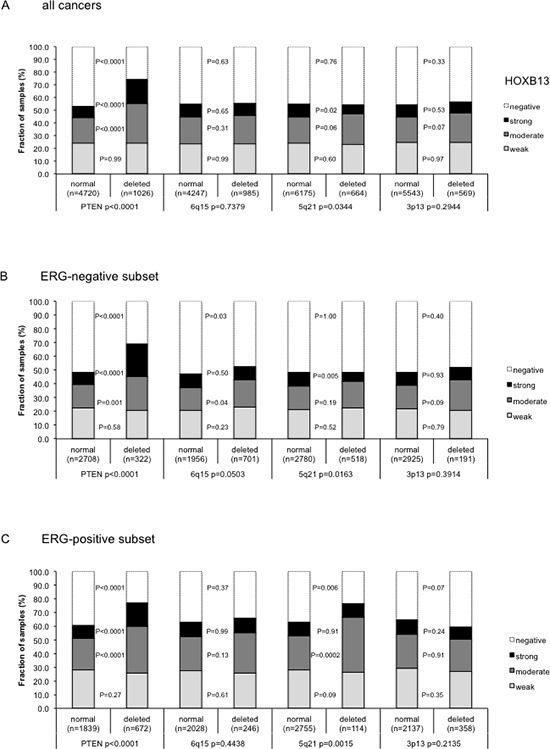
Association between TYMS immunostaining and 3p13, PTEN, 5q21, and 6q15 deletion in (a) all prostate cancers, (b) ERG negative cancers, and (c) in ERG positive cancers *P*-values next to the columns indicate the significance of differences between tumors with weak, moderate and strong staining. **(d)** Comparative analysis of TYMS expression in cancers without deletions of 3p, 5q, and 6q, and in cancers with one, two, or three of these deletions, in ERG negative and ERG positive cancer

Earlier studies had provided evidence for recurrent chromosomal deletions delineating further molecular subgroups amongst ERG positive and ERG negative prostate cancers. In particular, deletions of *PTEN* and 3p13 define subgroups of ERG positive, and deletions of 5q21 and 6q15 define subgroups in ERG negative cancers [20, 21]. To examine, whether TYMS expression might be particularly associated with one of these genomic deletions, TYMS data were compared to preexisting findings on *PTEN* (10q23), 3p13 (*FOXP1*), 6q15 (*MAP3K7*) and 5q21 (*CHD1*) deletions. The analysis revealed a strong association between high TYMS expression and 5q21 and 6q15 deletions (*p* < 0.0001, each) and a weaker association between high TYMS expression and 3p13 deletion (*p* = 0.0083), while TYMS expression was completely unrelated to PTEN deletion (Figure 3a). A comparative analysis of ERG positive and ERG negative cancers showed that these findings were largely similar in both groups even though somewhat more pronounced in ERG-negative cases (Figure 3b–3c). Remarkably, a combined analysis of the relationship between ERG or 3p/5q/6q deletions and TYMS expression revealed that the slight difference in TYMS expression was solely driven by deletions while there was no difference in TYMS expression between ERG positive and negative cancers without 3p/5q/6q deletions (*p* = 0.4474, Figure 3d). Among cancers with one, two, or all three of these deletions, there was a continuous increase of TYMS expression with the number of deletions (*p* < 0.0001, Figure 3d).

### Tumor phenotype and PSA recurrence

**Figure 4:**
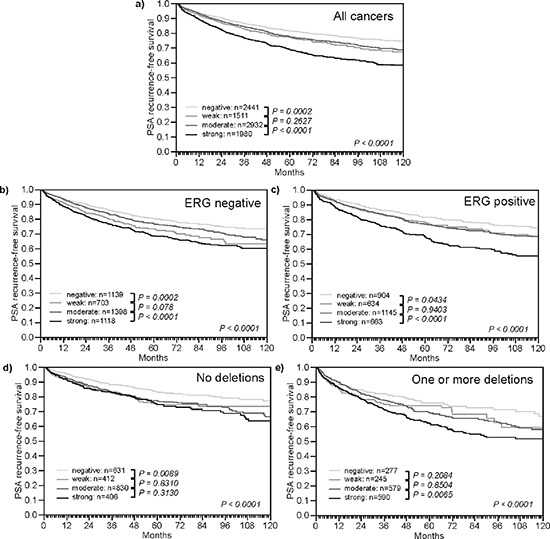
Impact of different levels of TYMS expression on PSA recurrence free survival in molecularly defined subsets of prostate cancer **(a)** All cancers. **(b)** Subset of ERG-fusion negative cancers. **(c)** Subset of ERG-fusion positive prostate cancers. **(d)** Subset of cancers without deletions of 3p13, 5q21, and 6q15. **(e)** Subset of cancers harboring one or more of these deletions.

High TYMS expression levels were associated with unfavorable tumor phenotype (Table 1). Significant associations were found with tumor stage, Gleason grade, preoperative PSA and surgical margin (*p* < 0.0001, each). High TYMS expression level was also associated with a higher risk for biochemical recurrence (Figure 4a, *p* < 0.0001). There was no major difference between ERG positive and ERG negative cancers in their relationship with PSA recurrence (Figure 4b–4c) and tumor phenotype (Supplementary Tables 2–3). Because of the strong link between TYMS and deletions of 3p, 5q, and 6q - which are all linked to poor prognosis [20, 21, 23] - separate subgroup analyses were also performed in cancers without these deletions (Figure 4d–4e), as well as in cancers harboring at least one of the three deletions (Figure 4e). The analysis revealed, that the prognostic value of high TYMS expression was independent from these deletions.

**Table 1:** Associations between TYMS immunostaining and clinico-pathological parameters of prostate cancer Chi^2^ *P*-values indicate the overall significance of associations across the categories.

		TYMS IHC result				
	*n* evaluable	negative (%)	weak(%)	moderate (%)	strong (%)	*P*-value
**All cancers**	10,223	27.1	17.6	33.4	21.9	
**Tumor stage**						
pT2	6,705	29.2	17.2	34.3	19.3	*< 0.0001*
pT3a	2,235	22.6	18.6	32.7	26.1	
pT3b	1,180	22.9	18.4	30.4	28.3	
pT4	59	37.3	11.9	23.7	27.1	
**Gleason grade**						
≤ 3 + 3	2,540	36.6	14.3	34.8	14.3	*< 0.0001*
3 + 4	5,720	25.1	19.0	34.5	21.4	
4 + 3	1,462	19.2	18.1	29.8	33.0	
≥ 4 + 4	444	22.1	17.6	24.1	36.3	
**Lymph node metastasis**						
N0	5,621	24.8	17.9	33.0	24.2	*0.0446*
N+	529	21.4	19.3	30.4	28.9	
**Preop. PSA level (ng/ml)**						
< 4	1,281	33.1	16.5	33.3	17.0	*< 0.0001*
4–10	6,148	27.0	18.1	34.5	20.4	
10–20	1,998	23.9	17.4	31.5	27.2	
> 20	675	23.6	16.0	30.5	29.9	
**Surgical margin**						
negative	8,196	27.8	17.9	33.6	20.6	*< 0.0001*
positive	1,840	23.7	16.0	32.3	28.0	

### Multivariate analysis

Four multivariate analyses were performed evaluating the clinical relevance of TYMS expression in different scenarios (Table 2). No. 1 utilized all post-operatively available parameters including pT, pN, margin status, pre-operative PSA value and Gleason grade obtained on the resected prostate. Scenario 2 utilized all postoperatively available parameters with the exception of nodal status. The rational for this approach was that lymphadenectomy is not a routine procedure in the surgical therapy of prostate cancer and that excluding pN in multivariate analysis increases case numbers. The next two scenarios tried simulate the pre-operative situation. Scenario 3 included TYMS expression, pre-operative PSA, clinical stage (cT) and the Gleason grade obtained on the prostatectomy specimen. Because the post-operative Gleason grade varies from the pre-operative Gleason grade, another multivariate analysis was added as scenario 4. In this scenario, the pre-operative Gleason grade obtained on the original biopsy was combined with pre-operative PSA, clinical stage and TYMS expression. The results identified TYMS expression an independent prognostic marker in the full study population in all 4 scenarios. TYMS did not reach independent prognostic impact in several preoperative scenarios in the (smaller) ERG positive and negative subgroups, however.

**Table 2:** Multivariate analysis of TYMS expression in all cancers, *ERG*-negative and *ERG*-positive cancers *P* – values correspond to Cox-regression analysis including different combinations of pre- and postsurgical parameters and TYMS expression.

*p*-value										
Tumor subset	Scenario	*n* analyzable	preoperative PSA-Level	pT Stage	cT Stage	Gleason grade prostatectomy	Gleason grade biopsy	N-Stage	R-Status	TYMS Expression
**All cancers**	1	5,552	*<0.0001*	*<0.0001*	-	*<0.0001*	-	*0.0001*	*0.0015*	*0.0325*
	2	9,235	*<0.0001*	*<0.0001*	-	*<0.0001*	-	-	*<0.0001*	*0.0079*
	3	9,110	*<0.0001*	-	*<0.0001*	*<0.0001*	-	-	-	*0.0243*
	4	8,986	*<0.0001*	-	*<0.0001*	-	*<0.0001*	-	-	*<0.0001*
**ERG-negative cancers**	1	2,846	*0.0002*	*<0.0001*	-	*<0.0001*	-	*0.0001*	*0.3301*	*0.2422*
	2	4,589	*<0.0001*	*<0.0001*	-	*<0.0001*	-	-	*0.0089*	*0.2897*
	3	4,556	*<0.0001*	-	*<0.0001*	*<0.0001*	-	-	-	*0.2625*
	4	4,501	*<0.0001*	-	*<0.0001*	-	*<0.0001*	-	-	*0.0089*
**ERG-positive cancers**	1	2,143	*0.0058*	*<0.0001*	-	*<0.0001*	-	*0.0459*	*0.007*	*0.2591*
	2	3,480	*<0.0001*	*<0.0001*	-	*<0.0001*	-	-	*<0.0001*	*0.0144*
	3	3,402	*<0.0001*	-	*<0.0001*	*<0.0001*	-	-	-	*0.0116*
	4	3,349	*<0.0001*	-	*<0.0001*	-	*<0.0001*	-	-	*0.0042*

## DISCUSSION

The results of our study identify high TYMS expression as a strong prognostic feature in prostate cancers, which is strikingly linked to certain chromosomal deletions.

Immunohistochemical experiments revealed positive TYMS staining in 73% of 10,223 prostate cancers. Two earlier studies analyzing 52 [12] and 79 [15] prostate tumors by immunohistochemistry had reported TYMS positivity in 67% [12] and in 100% [15] of cancers. These studies had analyzed either conventional large sections or TMAs with at least three 1.0 mm cores per cancer. As the frequency of TYMS expression detected in our study using TMAs constructed from one 0.6 mm core per patient is within the range of data reported from earlier studies using larger tissue quantities per patient, a methodological restriction related to our TMA approach is unlikely. Using the same TMA or smaller subsets of it, we had earlier reproduced the substantial prognostic impact of all previously well-established prognostic markers such as *PTEN* deletion [22], p53 alterations [24], chromosome 8q gains [25] or Ki67 labeling index [26]. We had also utilized this TMA platform for the successful identification of various other prognostic features in prostate cancer [19, 27–29]. TYMS immunostaining was more frequently and more strongly detected in prostate cancers than in normal prostate epithelium suggesting TYMS upregulation in a fraction of tumors. Our observation is in agreement with a study by Li et al. [12] also reporting higher TYMS expression in cancerous as compared to benign prostate tissues. However, Inoue et al. [15] suggested a reduced TYMS expression in prostate cancer.

TYMS upregulation was significantly associated with unfavorable tumor phenotype, rapid tumor cell proliferation, and early PSA recurrence in our patients. These findings are in line with earlier reports analyzing 52 [12] and 79 [15] prostate cancers by IHC or 172 cancers by RT-PCR [14]. These studies described significant associations between high TYMS expression and high Gleason score [12, 15] as well as advanced pathological tumor stage [12]. Two studies on 52 [12] and 88 [14] patients with follow-up data even described an association of high TYMS levels with biochemical recurrence. An unfavorable prognosis in prostate cancers with high TYMS expression is not unexpected based on the prognostic impact of TYMS found in various other tumors [7–10] and also based on the established functions of TYMS. Various other genes required for DNA synthesis and structural integrity have been shown to play a role in cancer development and progression, such as for example genes involved in nucleotide synthesis, including thymidine kinase 1 (TK1) [30] and ribonucleotide reductase M1 (RRM1) [31], genes involved in DNA synthesis such as DNA polymerases POLA1 [32], POLQ [33], and POLE [34], topoisomerases TOPO1 [35], TOP2A [36], and TOP3B [37], DNA ligases LIG3 [38] and LIG4 [39], as well as genes involved in maintenance of DNA structure and integrity, including helicases ATRX [40], FANCA-Q [41], RECQL1 [42], BRIP1 [43], HELQ [44], and chromatin modifiers like CHD1 [20] and CHD5 [45].

Recurrent chromosomal aberrations are defining prostate cancer subgroups. About 50% of prostate cancers undergo a TMPRSS2-ERG fusion [46]. Depending on whether or not this fusion is present, cancer cells are likely to develop specific chromosomal deletions. ERG activated cancers are most likely to develop deletions of PTEN or 3p13 while ERG negative cancers often acquire deletions of 6q or 5q [21, 22, 47–49]. Increased androgen signaling is a likely cause for development of ERG fusions [50]. The events that selectively favor the development of certain deletions are unclear. The combined analysis of ERG fusion and chromosomal deletion data in our large patient cohort strongly link TYMS overexpression to 6q, 5q and to a lesser extent to 3p deletions while TYMS is largely unrelated to PTEN deletions. The association with “non-ERG” deletions 5q and 6q was so strong that a significant statistical association between TYMS expression and negative ERG status was observed (Figure 2). This association completely vanished after correction for 5q/6q/3p deletions (Figure 3d). The specific association of TYMS expression with some but not all deletions is of interest because TYMS plays a role in DNA repair and as such may be mechanistically involved in deletion development. It might be speculated that one specific aspect of TYMS function is relevant for either development of some but not all deletions or alternatively that some deletions are of a specific kind prompting TYMS overexpression. This latter scenario is to some extent supported by the tendency towards an even higher TYMS expression in cancers with co-deletion of 3p, 5q or 6q. As an alternative, our findings could also be explained by a model suggesting that 5q, 6q and 3p harbor genes that suppress TYMS expression and that partial inactivation of one or more of these genes would trigger increased TYMS levels. However, we are not aware of genes that would be obvious candidates for a TYMS-suppressing role on 5q or 6q, and particularly not on 3p where deletions involve less than 10 genes in most cases [23, 49].

Deletions of 3p13, 5q21, and 6q15 have strong prognostic impact in prostate cancer [20, 21, 23]. It is thus of note that TYMS overexpression remains a strong prognostic feature, even if the analysis was limited to cancers harboring one or more of these deletions. Moreover, that the prognostic impact of TYMS was independent of established clinical and pathological prognostic features in various models of multivariate analysis applied in this study, further suggests a potential diagnostic utility of TYMS protein measuring in prostate cancer. This notion is also corroborated by the fact, that our approach of analyzing molecular features on a minute TMA tissue specimen, measuring 0.6 mm in diameter, closely models the molecular analyses of core needle biopsies where comparable amounts of tissues are evaluated. It is of note that the use of prostatectomy samples for the analysis of prognostic features has conceptual limitations as the natural history of these tumors is interrupted by potentially curative surgery, at least in many cases. Prostatectomy tissues are the only realistically available tissue sources for studies evaluating the prognostic impact of biomarkers, however. Molecular features can hardly be analyzed on preoperative biopsies because these are typically distributed among many different sites where the initial diagnosis was made and even if these biopsies were available for analyses such precious collection of tissues would be used up after only a few studies.

The rapidly increasing evidence for a relevant role of TYMS in cancer is also of potential therapeutic interest since some of the widely used cytotoxic drugs including 5-fluorouracil (5-FU) and capecitabine exert their anticancer effects by inhibiting TYMS [51–53]. 5-FU has been used alone or in combination with other chemotherapeutic drugs as palliative therapy for patients with advanced castration resistant prostate cancers [52, 54, 55]. Although 5-FU belongs to the most effective cytostatic agents, response rates in prostate cancer do typically not exceed about 20% [55]. Currently, there is no predictive biomarker of prostate cancer sensitivity to 5-FU. Several studies performed on other cancer types have proposed that high-level TYMS expression may predict success of 5-FU based chemotherapy [56–59]. Our findings encourage further studies to compare the efficacy of FU-based chemotherapy in prostate cancers and TYMS expression levels of the respective cancers.

In summary, our study identifies high TYMS expression as a strong and independent prognostic feature in prostate cancer, which is tightly linked to certain chromosomal deletions. TYMS expression analysis (either alone or in combination with other molecular parameters) might result in clinically useful information in prostate cancer.

## SUPPLEMENTARY TABLES



## Abbreviations

TYMS: Thymidylate synthase
ERG: V-Ets Avian Erythroblastosis Virus E26 Oncogene Homolog
PTEN: Phosphatase And Tensin Homolog
PSA: Prostate-specific antigen
DNA: deoxyribonucleic acid
pT: primär Tumor
pN: Lymph Node
dTTP: desoxy thymidine triphosphate
FISH: Fluorescence in situ hybridization
IHC: Immunohistochemistry
RT-PCR: Reverse transcription polymerase chain reaction
TK1: Thymidine kinase1
RRM1: Ribonucleotide reductase
POLA1: DNA Polymersase alpha 1
POLQ: DNA Polymersase theta
POLE: DNA Polymersase epsilon
TOPO1: Topoisomerase 1
TOP2A: Topoisomerase 2 alpha
TOP3B: Topoisomerase 3 beta
LIG3: DNA Ligase 3
LIG4: DNA Ligase 4
ATRX: Alpha Thalassemia/Mental Retardation Syndrome X-Linked
FANCA-Q: Fanconi Anemia, Complementation Group Q
RECQL1: DNA Helicase Q1-Like
BRIP1: BRCA1 Interacting Protein C-Terminal Helicase 1
HELQ: Helicase, POLQ-Like
CHD1: Chromodomain Helicase DNA Binding Protein 1
CHD5: Chromodomain Helicase DNA Binding Protein 5
TMPRSS: Transmembrane Protease, Serine
5-FU: 5-flourouracil
