# Supplementary material for: Overexpression of thymidylate synthase (TYMS) is associated with aggressive tumor features and early PSA recurrence in prostate cancer
Source: Oncotarget. 2015 Feb 25;6(10):8377–87. doi: 10.18632/oncotarget.3107 (PMC4480759; doi:10.18632/oncotarget.3107)
Supplement: Supplementary file 1 [file oncotarget-06-8377-s001.pdf]

SUPPLEMENTARY TABLES

Supplementary Table 1: Composition of the prognosis tissue microarray containing 11,152 prostate cancer specimens

	No. of patients	
	Study cohort on tissue microarray (*n* = 11,152)	Biochemical relapse among categories (*n* = 1,824)
**Follow-up (mo)**		
Mean	53.4	-
Median	36.8	-
Age (y)		
<50	318	49
50–60	2,768	460
60–70	6,548	1,081
>70	1,439	232
**Pretreatment PSA (ng/ml)**		
<4	1,407	142
4–10	6,735	827
10–20	2,159	521
>20	720	309
**pT category (AJCC 2002)**		
pT2	7,370	570
pT3a	2,409	587
pT3b	1,262	618
pT4	63	49
**Gleason grade**		
≤3 + 3	2,859	193
3 + 4	1,565	573
4 + 3	6,183	849
≥4 + 4	482	208
**pN category**		
pN0	6,117	1,126
pN+	561	291
**Surgical margin**		
negative	8,984	1,146
positive	1,970	642

NOTE: Numbers do not always add up to 11,152 in the different categories because of cases with missing data.
Abbreviation: AJCC, American Joint Committee on Cancer.

Supplementary Table 2: Clinico-pathological association of TYMS immunostaining in ERG fusion negative cancers

		TYMS IHC result				
	n evaluable	negative (%)	weak (%)	moderate (%)	strong (%)	*p* value
**All cancers**	5,083	25.7	16.5	32.7	25.1	
**Tumor stage**						
pT2	3,425	27.8	15.9	33.4	22.9	*<0.0001*
pT3a	1,023	21.3	17.2	31.7	29.8	
pT3b	593	21.1	18.7	30.7	29.5	
pT4	26	30.8	11.5	23.1	34.6	
**Gleason grade**						
≤3 + 3	1,160	37.2	14.1	33.0	15.7	*<0.0001*
3 + 4	2,831	23.8	17.7	34.5	24.1	
4 + 3	794	18.1	15.7	28.1	38.0	
≥4 + 4	276	19.2	16.3	26.1	38.4	
**Lymph node metastasis**						
N0	2,898	23.7	16.8	31.9	27.5	*0.173*
N+	262	20.2	19.5	28.2	32.1	
**Preop. PSA level (ng/ml)**						
<4	539	32.8	15.2	33.0	18.9	*<0.0001*
4–10	3,008	25.8	16.9	34.2	23.1	
10–20	1,107	22.9	16.3	30.2	30.7	
>20	382	22.0	14.9	28.5	34.6	
**Surgical margin**						
negative	4,074	26.4	16.8	33.1	23.6	*<0.0001*
positive	918	22.7	14.2	30.4	32.8	

Supplementary Table 3: Clinico-pathological association of TYMS immunostaining in ERG fusion positive cancers

	n evaluable	TYMS IHC result				*p* value
		negative (%)	weak (%)	moderate (%)	strong (%)	
**All cancers**	3,852	26.5	19.8	34.3	19.3	
**Tumor stage**						
pT2	2,317	28.8	19.7	35.6	15.9	*<0.0001*
pT3a	1,018	22.5	21.0	33.8	22.7	
pT3b	476	23.3	18.5	29.6	28.6	
pT4	23	30.4	17.4	26.1	26.1	
**Gleason grade**						
≤3 + 3	875	34.7	15.3	37.6	12.3	*<0.0001*
3 + 4	2,279	25.1	21.1	34.5	19.3	
4 + 3	546	20.0	21.6	31.7	26.7	
≥4 + 4	128	21.9	21.1	19.5	37.5	
**Lymph node metastasis**						
N0	2,145	24.4	20.0	34.2	21.4	*0.0446*
N+	222	22.5	19.8	32.0	25.7	
**Preop. PSA level (ng/ml)**						
<4	525	32.2	19.2	31.6	17.0	*<0.0001*
4–10	2,364	26.1	20.6	34.7	18.6	
10–20	678	22.9	19.2	34.8	23.2	
>20	233	24.9	17.2	36.1	21.9	
**Surgical margin**						
negative	3,034	27.4	20.2	34.2	18.2	*<0.0001*
positive	748	23.5	18.9	34.1	23.5	
